# Research hotspots and trends of non-invasive vagus nerve stimulation: a bibliometric analysis from 2004 to 2023

**DOI:** 10.3389/fneur.2024.1429506

**Published:** 2024-09-19

**Authors:** Mingyue Chen, Chunlan Yang, Yin Chen, Kailu Nie, Tingting Wang, Yun Qu

**Affiliations:** ^1^Department of Rehabilitation Medicine, West China Hospital of Sichuan University, Chengdu, Sichuan, China; ^2^College of Rehabilitation Medicine, West China Hospital of Sichuan University, Chengdu, Sichuan, China; ^3^Sichuan Provincial Key Laboratory of Rehabilitation Medicine, Sichuan University, Chengdu, Sichuan, China

**Keywords:** non-invasive vagus nerve stimulation, transcutaneous vagus nerve stimulation, neuromodulation, inflammation, bibliometric

## Abstract

**Objectives:**

Non-invasive vagus nerve stimulation (nVNS) is an emerging neuromodulation technique in recent years, which plays a role in nervous system diseases, psychiatric diseases, and autoimmune diseases. However, there is currently no comprehensive analysis of all the literature published in this field. Therefore, in this article, a bibliometric analysis will be conducted on all the literature published in the field of nVNS in the past 20 years.

**Methods:**

All articles and reviews published in this field from 2004 to 2023 were extracted from the WOS core database. VOSviewer 1.6.18.0, Scimago Graphica, CiteSpace 6.2.R2, and Excel 2021 were used to analyze the number of publications, participating countries, institutions, authors, references, and research hotspots in this field.

**Results:**

A total of 843 articles were included in the bibliometric analysis of nVNS. Over the past 20 years, the number of publications in this field has gradually increased, reaching a peak in 2023. The United States and China ranked top two in terms of publication volume, and institutions from these two countries also ranked high in terms of publication volume, citation count, and collaboration intensity. Rong Peijing is the author with the most publications, while Bashar W Badran is the most cited author. Articles in the field of nVNS were most frequently published in Frontiers in Neuroscience, while Brain Stimulation had the most citations. Currently, research hotspots in nVNS mainly focus on its application in diseases and related mechanisms.

**Conclusion:**

We conducted a comprehensive analysis of the field of nVNS, clarifying the previous research directions, which is helpful to expand its indications and promote clinical application.

## 1 Introduction

The vagus nerve is the X cranial nerve, part of the autonomic nervous system, and its function is to control the flow of information to and from glands and internal organs (1). With the development of neuromodulation techniques, the regulation of vagus nerve has also become a therapeutic target (2). Currently, VNS has been approved by the FDA for the non-drug treatment of drug-resistant epilepsy, depression, and migraine, and has also been explored for the treatment of other conditions, such as autism, Alzheimer’s disease, disorders of consciousness, and post-stroke rehabilitation (3). The regulation of vagus nerve can be divided into invasive and non-invasive methods. Non-invasive vagus nerve stimulation is to regulate vagus nerve activity by stimulating the vagus nerve projection area on the body surface via the auricle (transauricular vagus nerve stimulation, taVNS) or the cervical (transcervical vagus nerve stimulation, tcVNS), which affects the electrical activity of the brain and the function of the autonomic nervous system (4, 5). Compared to invasive vagus nerve stimulation, non-invasive vagus nerve stimulation offers similar benefits while avoiding the risks and adverse events associated with implants, including surgery risks, implant infections, electrode migration, and the need for ongoing care (6). Non-invasive vagus nerve stimulation has made significant progress in both clinical practice and research. However, there has not been a comprehensive review of the published literature in this field.

Bibliometrics is a research method that uses specific software to analyze published information such as books, journal articles, etc., and visualize the relationships between them (7). Bibliometrics visually shows the academic output of a research field and has become one of the important research tools to obtain a general overview and explore new fields (8, 9). Therefore, this article aims to provide a systematic overview of the research in non-invasive vagus nerve stimulation.

## 2 Methods

### 2.1 Data collection

All data was extracted and downloaded from the Web of science Core Collection (WoSCC)^1^ on March 17, 2024. After discussion among researchers, the search strategy was designed as TS = (Non-invasive vagus nerve stimulation*) OR TS = (transcutaneous vagus nerve stimulation), and publications ranging from January 1, 2004 to December 31, 2023, were retrieved. For a comprehensive overview, we have selected only research and review articles, excluded other publications, and limited the language to English, with publications exported as full records and citations, and kept in plain text file format.

### 2.2 Data analysis and tools

Before conducting visual analysis, we cleaned, transferred, and integrated the data obtained from WoSCC. Synonyms were replaced, meaningless words were removed, and confirmation was conducted before replacing some disputed areas with sovereign states. In this study, we used VOSviewer 1.6.18.0, CiteSpace 6.2.R2, and Scimago Graphics to generate a visual map of the exported literature. VOSviewer is a free visual web analysis software developed at Leiden University in 2009, which is used to display literature networks and build web maps of journals, institutions, authors, keywords, etc. (10). On the basis of the VOSviewer data, further geographical analysis of countries and institutions is performed using the Scimago Graphica software. Citespace is another free bibliometric analysis software built by Dr. Chen based on WOS database, which can directly reflect the development trend of academic field. In this paper, Citespace is used for visual analysis of institutions, keywords and references, as well as the relationship between the citing journals and the cited journals. In addition, Microsoft Office Excel 2021 is used to analyze the trend of annual publication quantity.

## 3 Results

### 3.1 Flow chart and annual article publication

**Figure 1:**
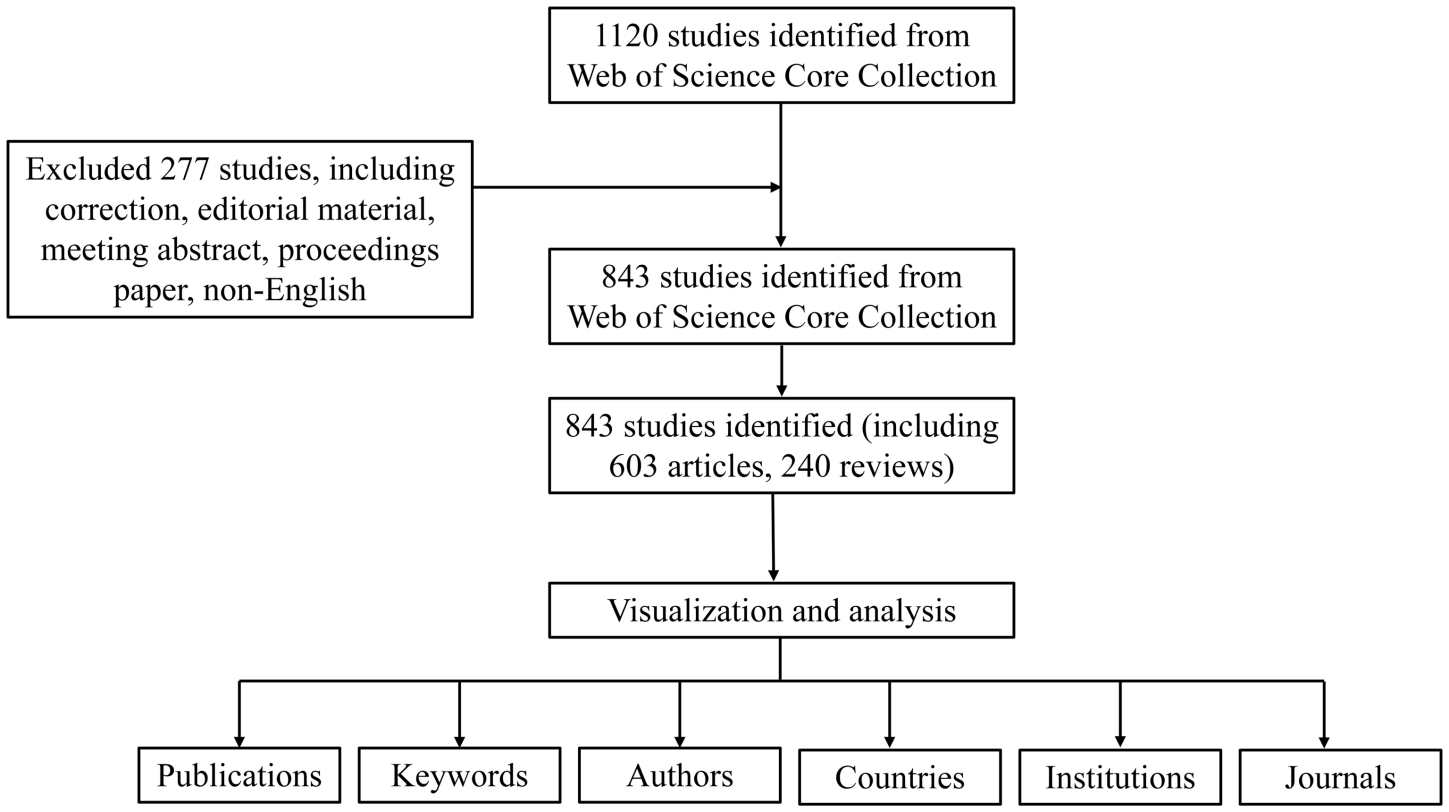
The flow chart of bibliometrics.

**Figure 2:**
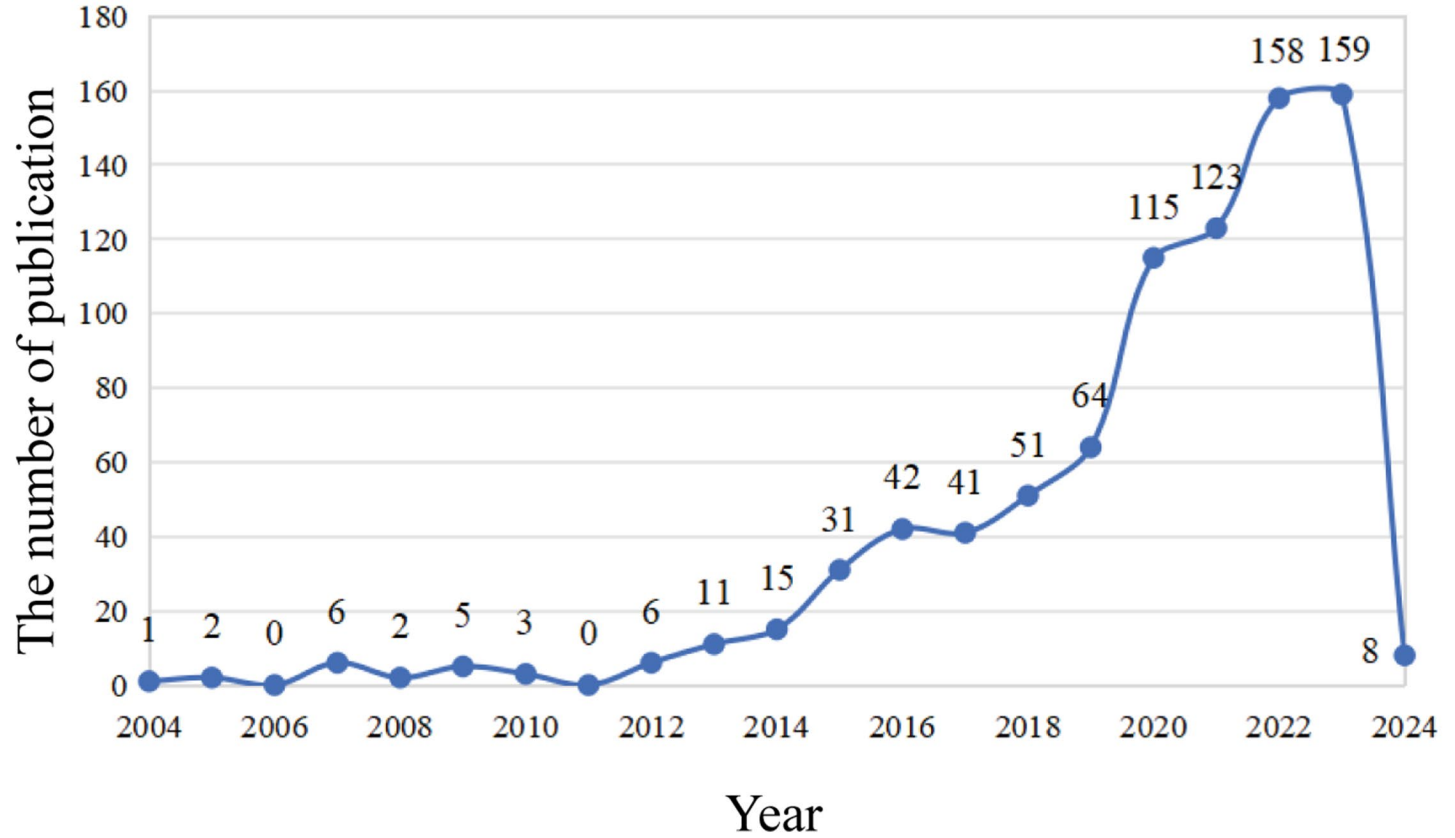
Annual publications non-invasive vagus nerve stimulation from 2004 to 2023.

As is shown in Figure 1, a total of 1,120 publications were retrieved in the WOS database, and 843 were imported into the software for visual analysis according to the exclusion criteria (Figure 1). In the past 20 years, the number of studies on the non-invasive vagus nerve stimulation has increased. It appears that there are distinct changes in the number of publications related to non-invasive vagus nerve stimulation (nVNS) in three specific years: 2013, 2015, and 2019 (Figure 2). Before 2013, the number of new studies on non-invasive vagus nerve stimulation was no more than 10 per year, and related publications increased year by year from 2015 to 2019. This uptick could be attributed to several factors, including advances in research methodologies, growing interest in the therapeutic potential of nVNS. Since 2020, the number of publications has reached more than 100 per year, and has already reached its peak in 2023 (Figure 2). This could reflect continued progress in the field, including the development of more refined techniques, improved understanding of the neurobiological mechanisms underlying nVNS, or the emergence of new clinical applications and indications for the therapy.

### 3.2 The analysis of countries and institutions

**Figure 3:**
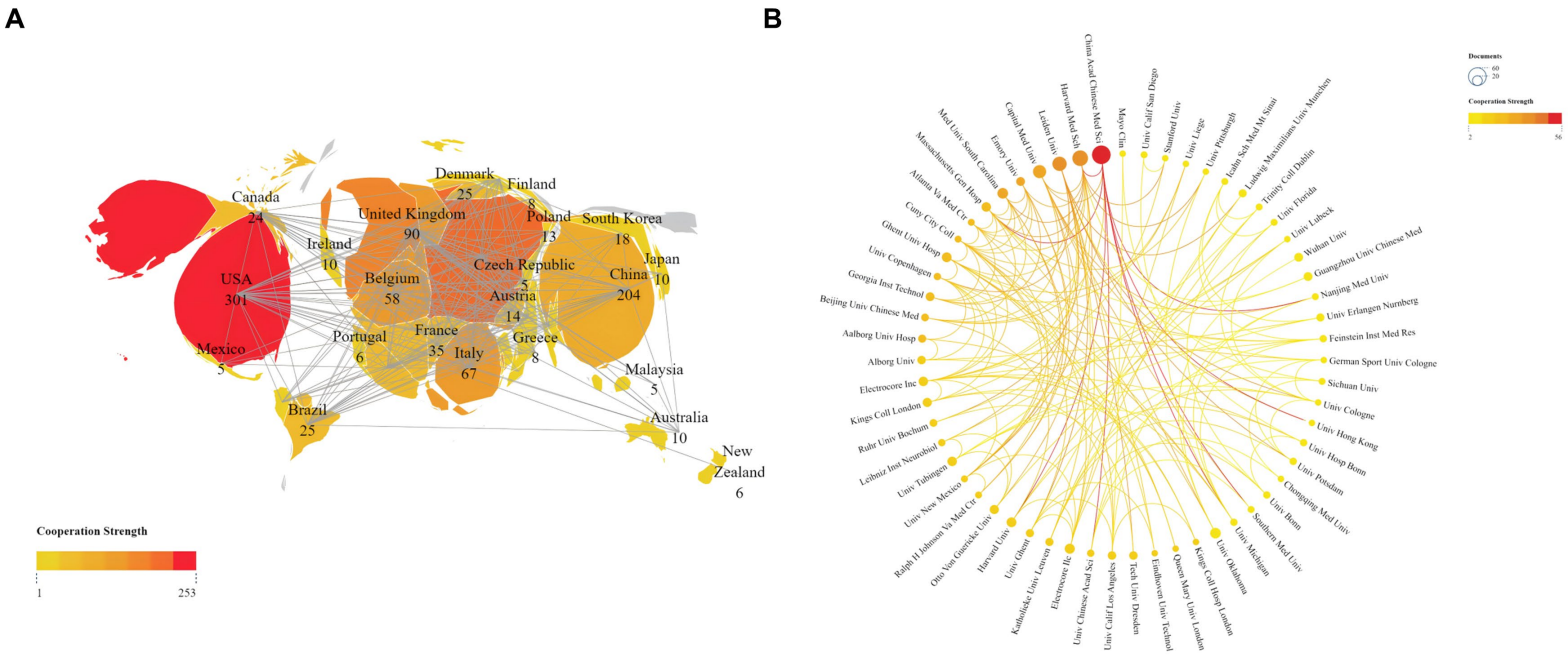
**(A)** Publications and cooperation networks of countries. **(B)** Publications and cooperation networks of institutions. The deeper the color of the connecting line, the stronger the cooperation intensity it represents.

We conducted a visual analysis using Vosviewer software, focusing on countries that have published at least five articles in the field of non-invasive vagus nerve stimulation (nVNS). From Figure 3A, we can observe a world map where the names of countries are accompanied by their respective publication counts. The brightness of the color representing each country indicates the level of collaboration with other nations. USA emerges as the leading country in terms of publication volume, followed by China, Germany, the United Kingdom, and Italy. This dominance is further underscored by the fact that the US also ranks first in citation counts and total link strength, indicating the significant impact and influence of American research in this area. While China closely follows the US in terms of publication output, its citation count ranks third, and its total link strength is in seventh place (Table 1A). This suggests that while Chinese scholars have made notable contributions to nVNS research, there is still room for further in-depth exploration. Additionally, it highlights the importance of strengthening collaborations with developed countries to promote the growth and advancement of this field. Other countries, such as Germany, the United Kingdom, and Italy, have published fewer articles than China but have higher citation counts and total link strengths. This indicates that while they may not have the high volume of output, their research is highly regarded and influential within the nVNS field. Subsequently, in Figure 3B, we conducted an analysis of 61 institutions that have published more than seven articles in the field of nVNS. Notably, the China Academy of Chinese Medical Sciences (CACMS) from China emerged as the institution with the largest publication volume (58) and the greatest total link strength ([TLS], 56). This suggests that nVNS may currently have more applications in traditional Chinese medicine. Meanwhile, the University of Erlangen-Nuremberg (Univ Erlangen-Nurnberg) from Germany received the most citations (1,638). Although their publication volume (11) is not as high, and their collaborations with other institutions are limited, their research in this field has still gained recognition from the academic community (Table 1B). This highlights the impact and significance of their work in advancing the understanding and application of nVNS.

**Table 1:** Counts, citations, and total link strengths (TLS) of publications of different countries and institutions.

A				
Rank	Country	Count number	Citations	TLS
1	USA	301	9,741	253
2	China	204	3,919	94
3	Germany	166	6,622	203
4	UK	90	3,377	156
5	Italy	67	2,054	115
6	Belgium	58	1,846	110
7	Netherlands	57	2,265	109
8	France	35	1,543	51
9	Spain	27	909	54
10	Denmark	25	1,406	41

B				
Rank	Institution (Country)	Count number	Citations	TLS
1	China Acad Chinese Med Sci (China)	58	1,448	56
2	Harvard Med Sch (USA)	41	1,127	37
3	Leiden Univ (Netherlands)	34	1,298	33
4	Electrocore Llc (USA)	32	1,673	28
5	Capital Med Univ (China)	29	541	32
6	Med Univ South Carolina (USA)	19	563	28
	Univ Oklahoma (USA)	19	783	9
7	Harvard Univ (USA)	16	1,486	13
	Ghent Univ Hosp (Belgium)	16	380	20
8	Univ Tubingen (Germany)	15	383	13
	Massachusetts Gen Hosp (USA)	15	546	23
9	Kings Coll London (UK)	14	530	15
	Otto Von Guericke Univ (Germany)	14	283	13
10	Emory Univ (USA)	13	148	30
	Georgia Inst Technol (USA)	13	97	19
	Guangzhou Univ Chinese Med (China)	13	200	5
	Wuhan Univ (China)	13	462	4

### 3.3 The analysis of co-authors and co-cited authors

**Figure 4:**
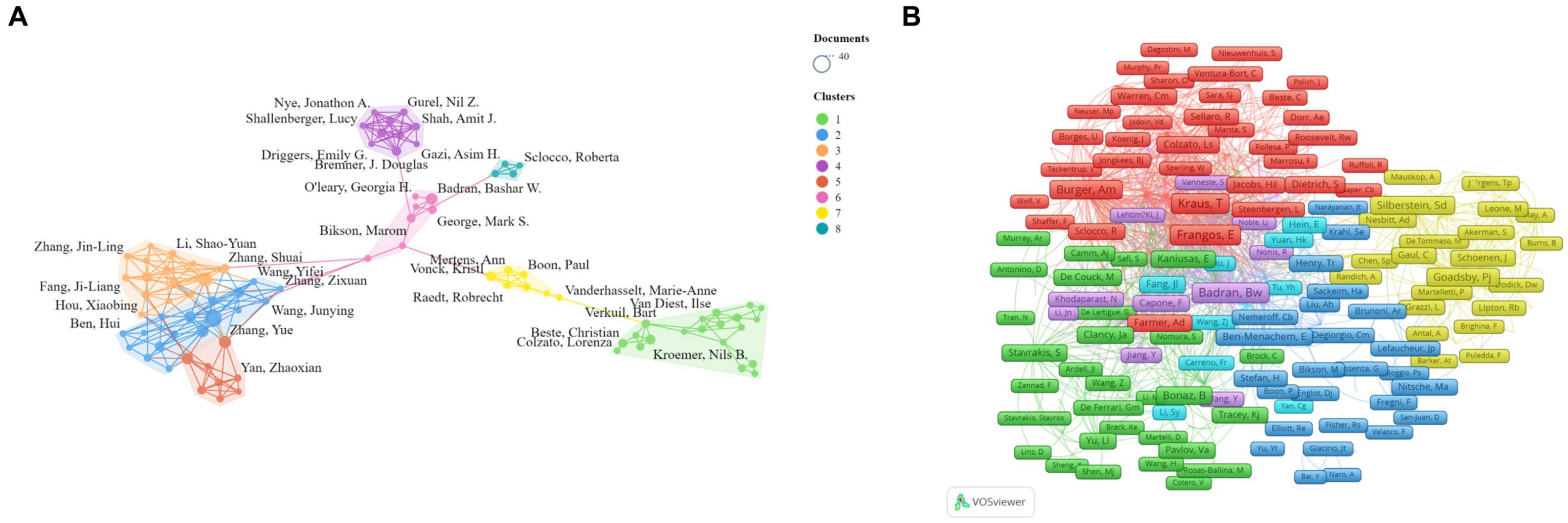
**(A)** Network visualization map of the co-authors. **(B)** Network visualization map of the co-cited authors.

We conducted a visual analysis of 89 authors with at least six publications in the field of non-invasive vagus nerve stimulation (nVNS) using Vosviewer and Scimago Graphica software. Chinese scholars have made significant contributions to the development of the field of non-invasive vagus nerve stimulation (nVNS). Professor Rong Peijing from the China Academy of Chinese Medical Sciences emerged as the author with the largest publication volume (36), citation count (1,146), and total link strength ([TLS], 135). Professor Wang Yu ranked second in terms of both publication volume and total link strength but did not rank as highly in citation count. Professors Kong Jian, Zhu Bing, Fang Jiliang, and Po, Sunny S. also ranked highly in terms of both publication volume and citation count in this field, indicating recognition from other researchers in the field. However, Professor Po, Sunny S. ([TLS], 23) could benefit from strengthening collaborations with others to foster better development (Figure 4A; Table 2). Additionally, Figure 4A reveals that cooperation among Chinese scholars is closer, with only a portion of them collaborating with international scholars. This suggests a need for further alignment with international research practices. We have also conducted an analysis of authors who have been co-cited at least 20 times, and 336 authors met this criterion. Our findings reveal that professors Badran BW, Krus T, Frangos E, and Burger AM are among the top five in terms of both citation count and total link strength (Figure 4B; Table 2). The high citation counts of these authors suggest that their research has been widely acknowledged and utilized by other researchers in the field, highlighting the impact and influence of their work. Additionally, their strong total link strengths indicate that they have established themselves as key figures within the research community, collaborating and influencing the work of others.

**Table 2:** Top 10 co-authors and co-cited authors related to non-invasive vagus nerge stimulation.

Rank	Co-author	Count number	Citation	TLS	Co-cited author	Citation	TLS
1	Rong, Peijing	49	1,289	245	Badran, BW	333	12,794
2	Wang, Yu	24	211	149	Kraus, T	306	10,037
3	Fang, Jiliang	18	720	94	Frangos, E	297	9,382
4	Badran, Bashar W	16	519	26	Burger, AM	253	12,442
5	Stavrakis, Stavros	15	617	27	Peuker, ET	225	6,435
6	Zhang, Jinling	13	150	84	Yakunina, N	213	7,734
7	Colzato, Lorenza S	12	399	17	Goadsby, PJ	210	6,574
8	Zaehle, Tino	11	164	5	Silberstein, SD	187	5,067
9	Goadsby, Peter J	10	589	11	Bonaz, B	171	5,014
10	Magis, Delphine	9	479	11	Rong, PJ	165	5,631

### 3.4 The analysis of journals and co-cited journals

**Figure 5:**
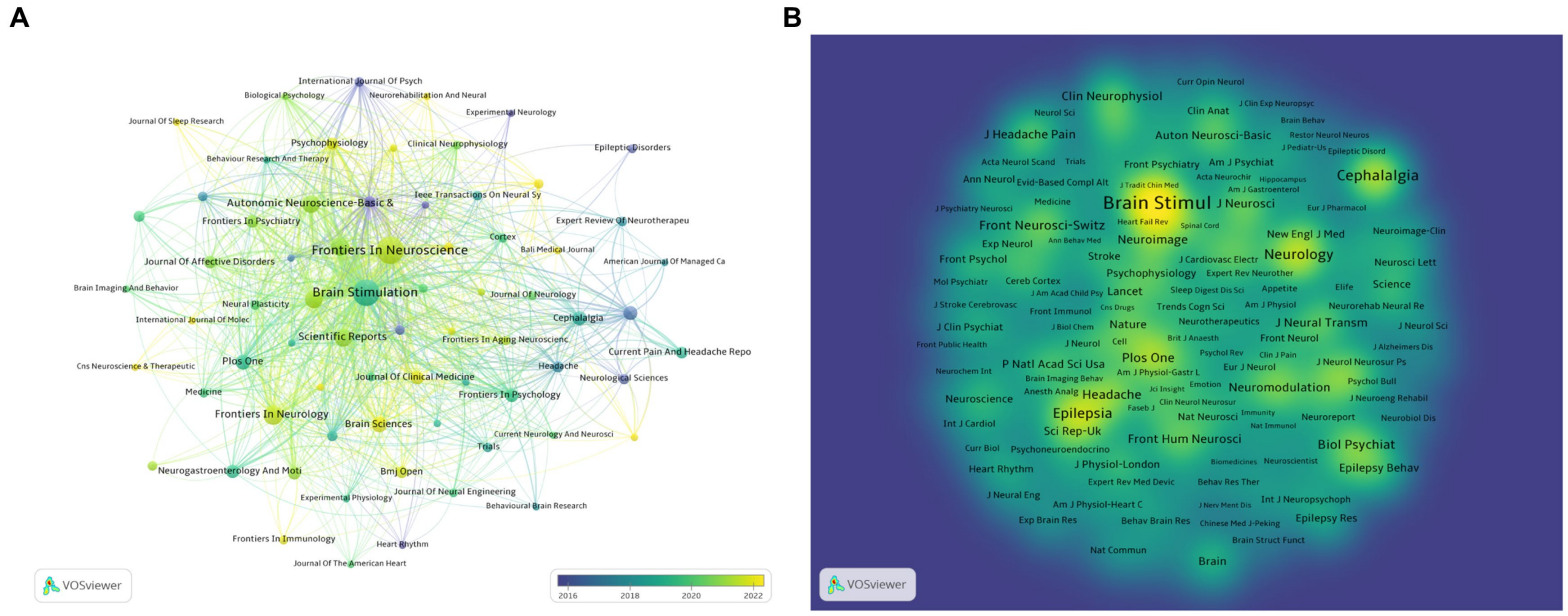
**(A)** Overlay visualization of journals. The lighter the color of the node, the closer the time of its appearance. **(B)** Density visualization of co-cited journals.

Using Vosviewer software, a visualization analysis was conducted on 70 journals that have published at least three articles on non-invasive vagus nerve stimulation (nVNS). The results revealed that in terms of publication volume, Frontiers in Neuroscience, Brain Stimulation, Frontiers in Neurology, Frontiers in Human Neuroscience, and Neuromodulation ranked in the top five (Figure 5A; Table 3). In terms of citation count and total link strength, Brain Stimulation emerged as the top-ranked journal, demonstrating its authority and influence in the field of nVNS. This indicates that the journal publishes high-quality research that is widely recognized and cited by other scholars in the field. Frontiers in Neuroscience ranked second in both citation count (713) and total link strength (673), indicating that it also plays a significant role in the field and has gained recognition from peers. The high citation counts and strong total link strengths of these journals suggest that they are key players in driving the development and dissemination of knowledge in nVNS. Overall, these findings can help researchers identify the most influential journals in the field and guide their publication and citation strategies accordingly. In addition, in Figure 5A, we can also observe changes in color, with lighter colors indicating more articles published in these journals in recent years, providing readers with a quick and intuitive way to identify journals that are actively publishing new research in the field of nVNS. In recent years, research in this field has shown a preference for publication in journals such as “Frontiers in Neuroscience,” “Frontiers in Psychiatry,” and “BMJ Open.” The visualization analysis of co-cited journals was also conducted using Vosviewer software. Figure 5B shows the analysis of 440 journals that have been co-cited more than 20 times. We found that Brain Stimulation stands out as the journal with the highest citation count and the strongest total link strength, significantly surpassing other journals in both aspects. The prominence of Brain Stimulation in this analysis underscores its importance and influence in the field of nVNS research. Its high citation count and strong total link strength indicate that it is a leading journal in the field, publishing cutting-edge research that is widely recognized and cited by other scholars. The journals that follow Brain Stimulation in terms of citation count and total link strength include Cephalalgia, Epilepsy, Brain Research, PLoS One, and Biological Psychiatry (Figure 5B; Table 3). This suggests that the current research on nVNS is likely to be more focused on areas such as headache, epilepsy, and other neurological and psychological disorders.

**Table 3:** Top 10 journals and co-cited journals related to non-invasive vagus nerge stimulation.

Rank	Journal	Count number	IF (2022)	JCR	Co-cited journal	Citation	IF (2022)	JCR
1	Frontiers in Neuroscience	46	4.2997	Q2	Brain Stimul	2,707	7.7002	Q1
2	Brain Stimulation	44	7.7002	Q1	Cephalalgia	1,265	4.9000	Q1
3	Frontiers in Neurology	23	3.4000	Q2	Neurology	1,188	9.8996	Q1
4	Frontiers in Human Neuroscience	21	2.8997	Q3	Epilepsia	1,058	5.5999	Q1
5	Neuromodulation	19	2.8002	Q2	Plos One	732	3.7001	Q2
6	Brain Sciences	14	3.3000	Q3	Headache	700	5.002	Q2
7	Plos One	13	3.7001	Q2	Biol Psychiat	685	10.6002	Q1
8	Cephalalgia	12	4.9000	Q1	Front Neurosci-Switz	680	4.2997	Q2
9	Journal Of Headache and Pain	11	7.4001	Q1	Neuromodulation	644	2.8002	Q2
10	Frontiers in Psychology	10	3.8001	Q2	Brain Res	627	3.3000	Q3

### 3.5 The analysis of keywords

**Figure 6:**
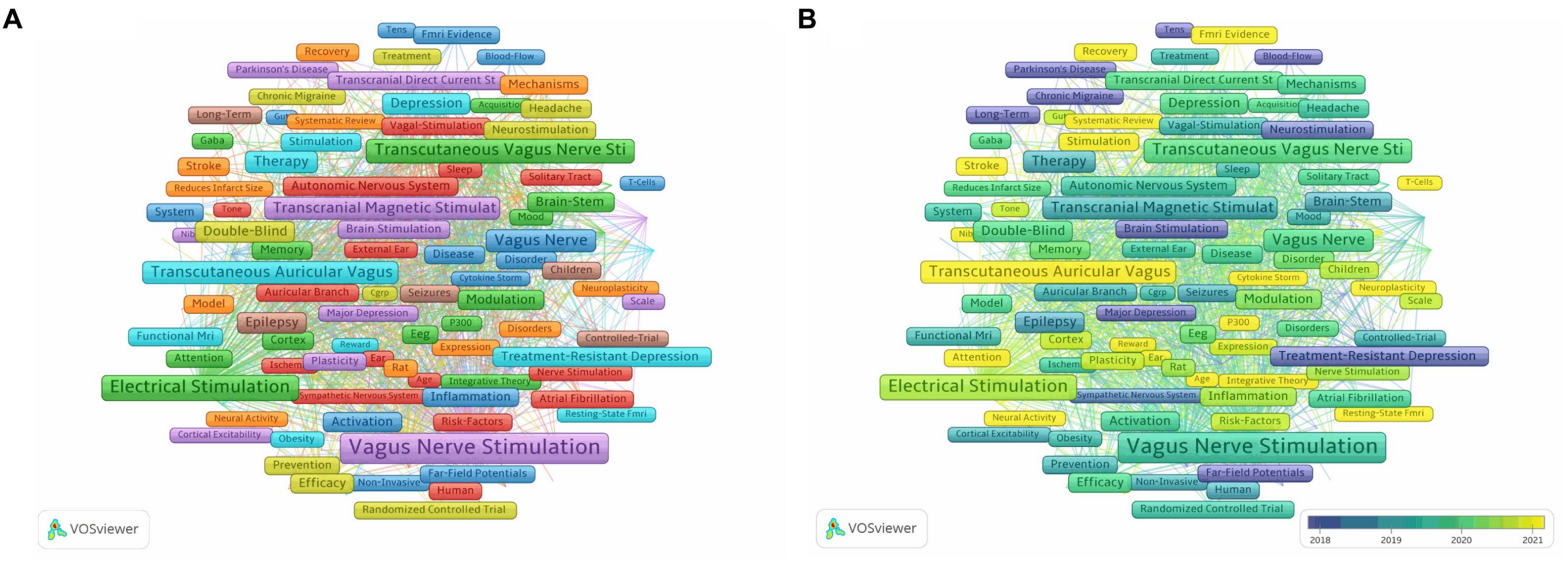
**(A)** Network visualization of keywords related to nVNS. **(B)** Overlay visualization of keywords related to nVNS. The lighter the color of the node, the closer the time of its appearance.

We conducted an analysis of 337 keywords that appeared at least 20 times. After manually ignoring keywords related to nVNS nomenclature, we found that the top 10 most frequently occurring keywords are Heart Rate Variability, Transcranial Magnetic Stimulation, Locus Coeruleus, Epilepsy, Double-Blind, Depression, Migraine, Treatment-Resistant Depression, Brain-Stem, and pain (Figure 6A). This indicates that current research on nVNS is focused on diseases such as epilepsy, depression, migraine, and pain. The stimulation sites, study designs, and differences from classic neuromodulation techniques of nVNS are also covered. At the same time, from Figure 6A, it can be observed that all the keywords can be grouped into eight clusters, which can be summarized into two main aspects. Firstly, the red, yellow, and light and dark blue clusters represent diseases where nVNS is applied or explored, such as chronic migraine, atrial fibrillation, and so on. These clusters indicate the range of medical conditions where nVNS has shown potential or is currently being investigated. Secondly, the dark blue, orange, green, brown, and purple clusters represent mechanistic studies related to nVNS, including topics like chemokine storms, blood flow, neuroplasticity, T-cells, and more. These clusters highlight the research efforts aimed at understanding how nVNS works at a molecular or cellular level, which is crucial for its further development and optimization. As shown in Figure 6B, in recent years, keywords such as Fatigue, Cognitive Impairment, Ischemic Stroke, Decision-Making, Disorders of Consciousness, Functional Recovery, Neural Activity, Rehabilitation, Cortisol, and Pupillometry have emerged. This suggests that nVNS is now being applied to functional recovery after stroke and the treatment of cognitive impairments. Additionally, researchers have begun to recognize potential side effects associated with nVNS.

### 3.6 The analysis of co-cited references and reference bursts

**Figure 7:**
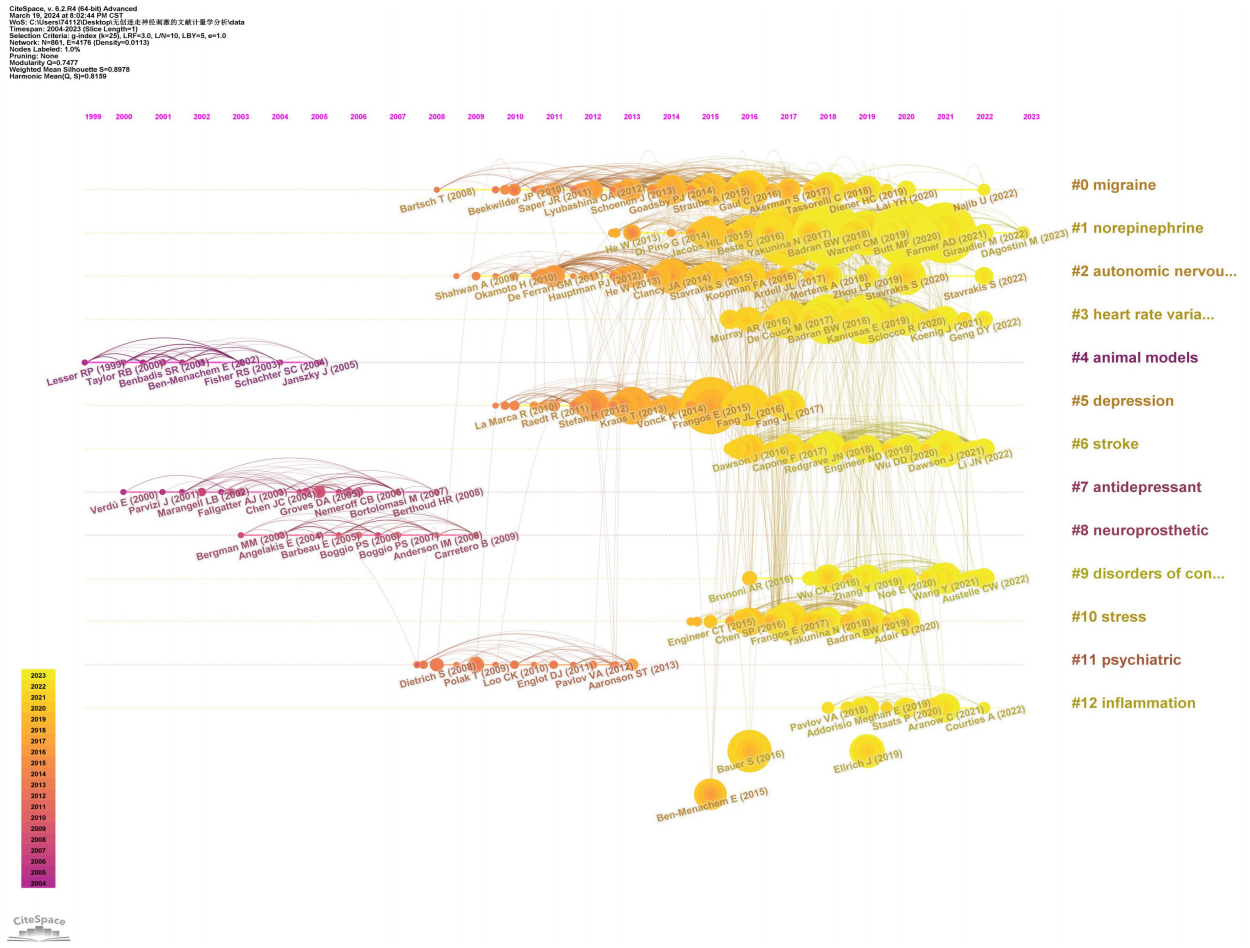
The timeline graph of the co-cited references.

We conducted a visualization analysis of the co-cited references in the field of nVNS based on keywords using Citespace software, generating a timeline graph (Figure 7). From Figure 7, we can see the literature that has been cited more frequently in each year, as well as the connections between the various cited literature. Examining the Figure 7, we can also observe 13 keywords listed on the right side, extracted from the co-cited references. From the timeline in the lower left corner of Figure 7, it can be seen that the more yellow the color, the closer the year of the cited literature in which the keyword appears. With the exception of “animal models,” “antidepressant,” “neuroprosthetic,” and “psychiatric,” the other nine keywords have emerged in the cited literature within the last decade. This trend indicates that the application of nVNS has gradually expanded from primarily focusing on psychiatric disorders to include neurological conditions such as stroke and disorders of consciousness. Simultaneously, research focused on the mechanisms of action and potential side effects of nVNS is also continuously increasing. Besides, as evident from the Table 4, the most central reference in this field is titled “Non-invasive access to the vagus nerve central projections via electrical stimulation of the external ear: fMRI evidence in humans,” published in Brain Stimulation (IF: 7.7002) in 2015. This groundbreaking paper, for the first time, demonstrated through functional magnetic resonance imaging (fMRI) that noninvasive vagus nerve stimulation via the auricular region produces neural projections consistent with those generated by “classic” vagus nerve stimulation (VNS). The publication of this paper coincided with a significant surge in the number of publications in the nVNS field, indicating its timeliness and impact.

**Table 4:** Top 25 references with the highest citation bursts related to non-invasive vagus nerve stimulation.

Rank	Author	Journal	DOI	Strength
1	Frangos E	Brain Stimul	10.1016/j.brs.2014.11.018	34.0953
2	Kraus T	Brain Stimul	10.1016/j.brs.2013.01.011	23.4471
3	Clancy JA	Brain Stimul	10.1016/j.brs.2014.07.031	18.4144
4	Stefan H	Epilepsia	10.1111/j.1528-1167.2012.03492.x	17.7741
5	Jacobs HIL	Neurobiol Aging	10.1016/j.neurobiolaging.2015.02.023	14.0671
6	Hein E	J Neural Transm	10.1007/s00702-012-0908-6	14.0104
7	Goadsby PJ	Cephalalgia	10.1177/0333102414524494	13.7852
8	Steenbergen L	Eur Neuropsychopharm	10.1016/j.euroneuro.2015.03.015	13.6509
9	Oshinsky ML	Pain	10.1016/j.pain.2014.02.009	13.3233
10	Silberstein SD	Headache	10.1111/head.12896	13.2784
11	Gaul C	Cephalalg	10.1177/0333102415607070	12.0256
12	Nesbitt AD	Neurology	10.1212/WNL.0000000000001394	11.6755
13	Barbanti P	Headache Pain	10.1186/s10194-015-0542-4	11.577
14	Bauer S	Brain Stimul	10.1016/j.brs.2015.11.003	11.5699
15	Straube A	Headache Pain	10.1186/s10194-015-0543-3	11.4617
16	Capone F	Neural Transm	10.1007/s00702-014-1299-7	10.6011
17	Busch V	Brain Stimul	10.1016/j.brs.2012.04.006	10.3646
18	Chen SP	Pain	10.1097/j.pain.0000000000000437	10.2206
19	Ben-Menachem E	Eur J Neurol	10.1111/ene.12629	10.1717
20	Rong PJ	J Affect Disorders	10.1016/j.jad.2016.02.031	10.1369
21	He W	Epilepsy Behav	10.1016/j.yebeh.2013.02.001	9.8445
22	Silberstein SD	Neurology	10.1212/WNL.0000000000002918	9.7054
23	Schoenen J	Neurology	10.1212/WNL.0b013e3182825055	9.5456
24	Fang JL	Biol Psychiat	10.1016/j.biopsych.2015.03.025	9.468
25	Butt MF	J Anat	10.1111/joa.13122	9.2435

## 4 Discussion

In this study, we conducted a bibliometric analysis of the field of noninvasive vagus nerve stimulation, providing a visual analysis of publication trends, countries, institutions, journals, major research authors, keywords, and references in this field. Over the past 20 years, the number of publications related to noninvasive vagus nerve stimulation has gradually increased, reaching a peak in 2023. This indicates that more researchers have recognized the importance and potential of this neuromodulation technique. Among the top 10 countries in terms of publication volume, China stands as the sole developing nation, contributing a significant amount of research output. In terms of research institutions, although the China Academy of Chinese Medical Sciences, a Chinese research institution, boasts the highest number of publications and overall citation strength, the other top 10 institutions are all from developed countries. Hence, research institutions in developing countries ought to enhance the depth of their research and foster collaboration. Our keyword analysis has revealed that recent research on noninvasive vagus nerve stimulation (nVNS) has primarily focused on its application in various diseases, potential mechanisms, and possible side effects. Therefore, this article aims to provide a comprehensive overview of these aspects.

### 4.1 The application and potential mechanism of non-invasive vagus stimulation in various diseases

#### 4.1.1 Depression

Initially, the antidepressant effect of vagus nerve stimulation (VNS) was discovered during the treatment of epilepsy. Several studies targeting epilepsy found that VNS could influence the levels of 5-hydroxyindoleacetic acid, the primary metabolite of 5-hydroxytryptamine (5-HT, also known as serotonin), and was subsequently recognized as having antidepressant properties (11). In 2005, the FDA approved the use of VNS for the treatment of major depressive disorder (12). However, due to the high cost and invasive nature of traditional VNS, non-invasive vagus nerve stimulation (nVNS) has gained attention in recent years (13). The study results indicate that the combination of non-invasive vagus nerve stimulation (nVNS) and antidepressant medication led to significant improvement in depressive symptoms among patients, with a reduction of 12.6 points in the Beck Depression Inventory (BDI) score compared to medication alone (14). When compared to other non-invasive neuromodulation techniques such as transcranial magnetic stimulation (TMS) and transcranial direct current stimulation (tDCS), nVNS demonstrated more pronounced improvement in depressive symptoms (15, 16). Another non-randomized controlled trial demonstrated that patients with major depressive disorder (MDD) treated with non-invasive vagus nerve stimulation (nVNS) experienced a significant reduction in their 24-item Hamilton Depression Rating Scale (HAMD) scores compared to those in the sham nVNS treatment group. The magnitude of improvement was notably greater in the nVNS group, and this clinical improvement persisted until the end of the study (17). The mechanism by which nVNS improves depression may be correlated with the pathogenesis of depression, including the modulation of functional connectivity in depression-related brain regions, regulation of neurotransmitters, modulation of the HPA axis, and amelioration of inflammation (18). Multiple evidences suggest that depression is associated with structural and functional abnormalities in various brain regions such as the subgenual cingulate cortex, anterior insula, hippocampus, and hypothalamus (19, 20). These regions are involved in emotional processing, self-representation, reward, and the interaction of external stimuli (stress, pain), nVNS can activate the activity and functional connectivity of these brain regions, thereby improving depressive symptoms (21–23). Dysregulation of neurotransmitters is also one of the mechanisms underlying depression, and studies have shown that nVNS can regulate the levels of neurotransmitters such as 5-HT, NE, DA, and glutamate to improve depressive symptoms (24, 25). The activation of the HPA axis, which is associated with stress responses, is a typical neurobiological alteration in patients with depression (26). nVNS can regulate the hormonal changes and stress responses caused by high activity of the HPA axis (27). Similarly, inflammatory responses are pathological changes that promote depressive symptoms, and nVNS can inhibit inflammatory responses to relieve stress and depressive symptoms (28). In addition, studies have shown that the gut microbiota in patients with MDD is disrupted (18). The gut microbiota can interact with the central nervous system through endocrine and immune pathways (29). Therefore, nVNS can regulate the brain-gut axis to achieve its therapeutic effect on depression (30).

#### 4.1.2 Epilepsy

Vagus nerve stimulation, as the first neurostimulation device approved by the FDA for the treatment of refractory epilepsy, has been implanted in more than 100,000 patients and has shown good tolerance (31). The results of some clinical trials have shown that the effective response rate of invasive vagus nerve stimulation is between 40 and 60%; however, the side effects of the invasive procedure have caused distress to patients (32, 33). Non-invasive vagus nerve stimulation has been proven to have a similar therapeutic effect on epilepsy as invasive stimulation (34, 35). Preliminary small-sample studies have indicated that non-invasive vagus nerve stimulation (nVNS) has a therapeutic effect on both pediatric and adult epilepsy. In pediatric epilepsy, the response rate for seizure control and the average reduction in seizure frequency can reach up to 53.85 and 54.21%, respectively (36, 37). Studies on the treatment of epilepsy with different frequencies of nVNS have shown that, compared to the low-frequency treatment group, patients in the high-frequency stimulation group who completed the full treatment period experienced a 34.2% reduction in seizure frequency (38). Extensive randomized controlled trial (RCT) results have demonstrated that the response rate in the non-invasive vagus nerve stimulation (nVNS) group is significantly higher than that in the sham stimulation group (38–40). Recent RCT findings reveal that the nVNS group exhibited a response rate of 44.74% at week 20, which was notably higher than the control group’s rate of 16.67%. There were no significant differences in response rates at weeks 4 and 12, suggesting that nVNS may require a prolonged treatment period to exert its therapeutic effect (41). The mechanisms of action of non-invasive vagus nerve stimulation (nVNS) in the treatment of epilepsy include the release of norepinephrine, alterations in cerebral blood flow within the brain, changes in functional connectivity of the brain, inhibition of certain key targets of excitatory proteins in the brain, and suppression of inflammatory responses (42). By stimulating the vagus nerve, nVNS initiates a cascade of neurochemical and physiological responses that modulate brain activity and reduce seizure susceptibility. The release of norepinephrine, a neurotransmitter, is thought to play a crucial role in seizure suppression by regulating neuronal excitability (40, 43). Additionally, nVNS-induced changes in cerebral blood flow may contribute to seizure control by optimizing oxygen and nutrient delivery to critical brain regions (44–46). Furthermore, studies have shown that nVNS can modulate functional connectivity between different brain regions, potentially aiding in the restoration of normal brain network activity and subsequently reducing epileptic seizures (43). The inhibition of excitatory proteins and inflammatory responses represent additional mechanisms by which nVNS exerts its anti-seizure effects (3, 47).

#### 4.1.3 Stroke

Stroke is a common emergency of the central nervous system that affects millions of people worldwide each year. The treatment and long-term rehabilitation of stroke impose a huge burden on both families and society. As a type of neuromodulation technique, nVNS plays a significant role in improving symptoms and facilitating long-term recovery for stroke patients. nVNS can help to modulate the nervous system’s activity, promoting neural plasticity and angiogenesis, potentially leading to improved recovery of motor, sensory, and cognitive functions after a stroke. This technique may also have a positive impact on reducing inflammation, regulating blood–brain barrier (BBB) permeability, regulating heart rate and blood pressure, and improving overall well-being, which are all important factors in the recovery process (1). A small preliminary trial recruiting 13 patients with residual upper limb dysfunction more than 3 months after ischemic stroke found that nVNS-assisted rehabilitation training can promote the recovery of upper limb function in patients, with significant changes in Fugl-Meyer scores (48). Another study has shown that nVNS combined with rehabilitation training can promote proprioceptive recovery in stroke patients with motor dysfunction. Moreover, patients with better motor function recovery tend to have more significant increases in their Fugl-Meyer sensory scores. This may suggest that the recovery of motor function after a stroke could potentially enhance the neuroplasticity of cortical sensory networks, forming a positive feedback loop between motor and sensory recovery (49). Furthermore, a case report demonstrates significant improvement in insomnia symptoms among stroke patients who underwent intensive nVNS treatment. The PSQI (Pittsburgh Sleep Quality Index) scores decreased notably, and the therapeutic effect was still observable 3 months after the treatment (50). A randomized, double-blind pilot study conducted by Badran BW in 2023, found that among patients with upper limb dysfunction after stroke, the Fugl-Meyer score increased more in the motor-activated auricular vagus nerve stimulation group compared to the group receiving only auricular vagus nerve stimulation (51). This may indicate that the therapeutic effect of auricular vagus nerve stimulation combined with motor is superior to that of non-invasive vagus nerve stimulation alone, pointing out a new path for the further development of non-invasive vagus nerve stimulation.

#### 4.1.4 Migraine

Migraine is the most common type of primary headache in clinical practice, often affecting women. It typically manifesting as recurrent episodes of moderate to severe headache on one or both sides of the head, and may be accompanied by symptoms such as nausea, vomiting, photophobia, and phonophobia. Migraine has a relatively high incidence rate, typically ranging from 5 to 15%. This means that in a specific population, approximately 5–15 out of every 100 individuals may experience migraine, causing significant distress to the patients. Currently, pharmacological treatments, including acute and preventive migraine medications, are commonly used for migraine intervention. However, due to suboptimal efficacy, contraindications, and side effects of migraine medications, the results of pharmacological treatment for migraine appear to be less than satisfactory. Therefore, there is a need to explore non-pharmacological treatment options. Non-invasive neuromodulation techniques such as single-pulse transcranial magnetic stimulation (s-TMS) and external trigeminal nerve stimulation (e-TNS) have been approved by the FDA for the prevention and acute treatment of migraine. Furthermore, as the therapy with the highest level of evidence in acute management of migraine, transcervical vagus nerve stimulation (tcVNS) has also received FDA approval in 2018 (52). The results of several clinical studies indicate that tcVNS can reduce the intensity of pain in migraine patients while decreasing the use of analgesic medications, particularly showing better efficacy in patients with migraine with aura (53, 54). In comparison, low-frequency non-invasive auricular vagus nerve stimulation (n-aVNS) can significantly reduce the number of migraine days and headache intensity (55, 56). Studies have also shown that continuous prophylactic use of tcVNS can reduce the number of headache days (57). Overall, non-invasive vagus nerve stimulation, regardless of the site, can alleviate the symptoms of migraine and help patients return to normal life (58). The mechanism of action of nVNS in the treatment of migraine is multifaceted and may involve various aspects such as autonomic nervous system regulation, cortical spreading depression, neurotransmitter modulation, and nociceptive information regulation. The vagus nerve is a crucial component of the autonomic nervous system. By stimulating the vagus nerve, the balance between the sympathetic and parasympathetic nervous systems can be modulated, thereby influencing vascular abnormalities and inflammatory responses associated with migraine. Some studies have suggested that migraine attacks are associated with cortical spreading depression (CSD), a wave-like propagation of neuronal and glial depolarization. Non-invasive vagus nerve stimulation may alleviate migraine symptoms by inhibiting the propagation of CSD (59). Similarly, nVNS may exert the same effect by modulating functional connectivity between different brain regions (56). Furthermore, vagus nerve stimulation affects the release of various neurotransmitters, such as acetylcholine, dopamine, and 5-hydroxytryptamine, which play crucial roles in the pathogenesis of migraine (60). By regulating the levels of these neurotransmitters, it may help to relieve migraine. Finally, during a migraine attack, nociceptive information is transmitted from the trigeminovascular system to the brain (34). Non-invasive vagus nerve stimulation may reduce the pain sensation of migraine by modulating the transmission and processing of this nociceptive information (61).

#### 4.1.5 Systemic lupus erythematosus and rheumatoid arthritis

Systemic lupus erythematosus (SLE) and rheumatoid arthritis (RA) are both chronic systemic autoimmune diseases with complex causes and unclear pathogenesis. Both diseases share similar treatments including nonsteroidal anti-inflammatory drugs, glucocorticoids and immunosuppressants. However, although the use of these drugs has greatly relieved the clinical symptoms of patients, it has brought some unavoidable side effects to patients, such as infection of different sites, gastrointestinal events, adverse reactions of cardiovascular and skeletal muscle systems. Therefore, non-drug treatment, especially vagus nerve stimulation, has attracted the attention of researchers. Studies have shown that vagus nerve stimulation can reduce the severity of rheumatoid arthritis and relieve clinical symptoms (62, 63). Since vagus nerve stimulation can cause some invasive injuries to patients, attention has begun to turn to non-invasive vagus nerve stimulation. The results of two open-label studies in 2021 showed that non-invasive vagus nerve stimulation can reduce the inflammatory response of rheumatoid arthritis, and DAS28-CRP had a clinically significant reduction (63, 64). In addition, Marsal et al. Found in their study that 16 patients (53%) achieved ACR20 response, 10 patients (33%) achieved ACR50 response, and five patients (17%) achieved ACR70 response, indicating that under the treatment of nVNS, the clinical symptoms of half of the patients were relieved by 20%, and the clinical symptoms of one third of the patients were relieved by 50% (63). A randomized double-blind sham-controlled trial of noninvasive vagus nerve stimulation for the treatment of systemic lupus erythematosus in 2021 found that compared with the control group, patients in the taVNS treatment group had significantly reduced pain and fatigue, and joint tenderness and swelling were also significantly improved. This effect lasted for about a week after the stimulation ended (65). Animal experiments have shown that non-invasive vagus nerve stimulation can reduce the number of hippocampal microglia by activating tyrosine hydroxylase-positive neurons in the locus coeruleus, thereby reducing central nervous inflammation. It can also delay peripheral lymph node expansion and splenomegaly, and reduce nephritis (66, 67). Currently, abnormal autoimmune response is considered to be the core mechanism of the pathogenesis. When autoimmunity is activated in SLE and RA patients, a series of proinflammatory molecules such as TNF-α, IL-6, IL-1β, IFN, free radicals, etc. are released, initiating and maintaining the inflammatory process, which may lead to some clinical symptoms of the disease (68). The mechanism of non-invasive vagus nerve stimulation in the treatment of autoimmune diseases may be related to the cholinergic anti-inflammatory pathway (CAP). The cholinergic pathway is a long loop starting from vagus nerve afferent, passing through the autonomic brainstem and forebrain cortical structures, and then returning via descending vagus nerve efferent (69). When the vagus nerve is activated, acetylcholine is released and binds to the α nicotinic receptor (α7nAChRs) expressed in macrophages, inhibiting the release of related inflammatory factors, thereby inhibiting the inflammatory response (5). In summary, we can see that non-invasive vagus nerve stimulation can alleviate the clinical symptoms of rheumatoid arthritis and systemic lupus erythematosus. However, there is currently limited high-level evidence for both diseases, and further evaluation is needed in larger-scale controlled studies.

#### 4.1.6 Cardiovascular disease

Cardiovascular diseases, with high morbidity and mortality rates, are one of the leading causes of death. They can be mainly divided into coronary heart disease, hypertension, heart failure, arrhythmia, cardiomyopathy, and heart valve disease (70). The dysfunction of the autonomic nervous system is correlated with the occurrence and progression of these diseases (71). As part of the autonomic nervous system, the descending cardiac branch of the vagus nerve is crucial for normal heart function, therefore modulation targeting the vagus nerve may be an effective measure for the treatment of these diseases (68). Previous studies have shown that high-intensity vagus nerve stimulation can induce heart failure, but low-level vagus nerve stimulation is believed to inhibit atrial fibrillation, and low-intensity taVNS has been proven to have the same effect. A study in 2013 first confirmed that LL-taVNS can reverse rap-induced atrial remodeling in canine models and inhibit the inducibility of atrial fibrillation (72). Subsequently, another study found that the mechanism of action may be the regulation of the expression of atrial connexin 40 and connexin 43 (73, 74). The results of a randomized controlled trial showed that LL-taVNS may inhibit the occurrence of postoperative atrial fibrillation by suppressing inflammatory responses (75). Similarly, taVNS can regulate the expression of collagen, TGF-β, and MMP-9 in chronic myocardial infarction models, maintain the stability of cardiac electrophysiology, thereby improving ventricular remodeling and function, and reducing the occurrence of ventricular arrhythmias (76–78). In heart failure with preserved ejection fraction, taVNS can reduce cardiac inflammatory response and cardiac fibrosis, improving diastolic dysfunction (79). Overall, non-invasive vagus nerve stimulation demonstrates a protective effect on certain cardiovascular diseases. The mechanism may be related to reducing inflammatory responses, decreasing oxidative stress, and regulating the balance of the cardiac autonomic nervous system. However, this protective effect is currently limited to the preclinical stage, and substantial clinical evidence is needed to promote the clinical application of non-invasive vagus nerve stimulation in the cardiovascular system.

### 4.2 Possible side effects

Current clinical studies have found that nVNS has good safety and tolerability. Compared to invasive vagus nerve stimulation, the most common adverse reactions are rash, pain, erythema, discomfort, tinnitus, and dizziness at the contact site, which can be resolved after discontinuing the treatment.

## 5 Conclusion

This study has certain limitations. Firstly, we only extracted English literature from the core database of WOS, which may result in insufficient comprehensive literature included in the study. In addition, we elaborated on the diseases that are currently studied more in nVNS, and did not summarize the less studied diseases, which needs further elaboration in subsequent studies. In summary, to our knowledge, this is the first bibliometric analysis specifically targeting non-invasive vagus nerve stimulation. We have organized the publication trends in this field, focusing on the key researching countries, institutions, and research hotspots, including related diseases, mechanisms of action, and more. These findings contribute to exploring new indications for non-invasive vagus nerve stimulation, promoting its further clinical application, and enhancing the clinical efficacy of disease treatment.

## Data Availability

The original contributions presented in the study are included in the article/supplementary material, further inquiries can be directed to the corresponding author.

## Footnotes

^1^ https://www.webofscience.com/wos/woscc/basic-search
